# Claudin-1 Interacts with CD81 and Promotes the Progression of Colorectal Cancer

**DOI:** 10.32604/or.2026.075185

**Published:** 2026-05-21

**Authors:** Kaidi Yin, Lili Deng, Wen Liu

**Affiliations:** Department of Radiology, Jinshan Hospital, Fudan University, Shanghai, China

**Keywords:** Colorectal cancer, claudin-1 (CLDN1), CD81, ubiquitination, migration, invasion

## Abstract

**Objectives:** Although claudin-1 (CLDN1) interacts with Cluster of Differentiation 81 (CD81) in various cell types, the specific mechanism underlying this interaction and its functional implications in colorectal cancer (CRC) cells remain poorly understood. This study outlines the regulatory role of CLDN1 in CRC cell tumorigenicity through its interaction with CD81, elucidating the underlying signaling cascade. **Methods:** Changes in the expression of CLDN1 and CD81, as well as their correlation with the survival of CRC patients, were analyzed using samples from The Cancer Genome Atlas database, the Kaplan‒Meier plotter database, and tissue microarrays. CLDN1 and CD81 were silenced in CRC cell lines to examine their effects on cell viability, migration, and invasion. The interaction between CLDN1 and CD81, as well as the regulation of CD81, was examined via coimmunoprecipitation and ubiquitination analysis. CLDN1-overexpressing SW620 cells and a xenograft tumor model were cotreated with the anti-CD81 monoclonal antibody (mAb) 5A6 to investigate the role of the CLDN1/CD81 axis in CRC tumor growth. **Results:** CLDN1 expression was enhanced in CRC tissue and was correlated with poor survival in patients. Analysis revealed a significant upregulation of CLDN1 in all examined CRC cell lines relative to normal intestinal epithelial controls. Silencing of CLDN1 and CD81 reduced the CRC cell viability, invasion and migration. CLDN1 interacted with CD81 and promoted CD81 expression by suppressing CD81 ubiquitination. The anti-CD81 mAb 5A6 reversed the functions of CLDN1 overexpression in CRC malignant phenotypes and tumor xenograft growth. **Conclusion**: This study establishes CLDN1 as a promising therapeutic target in CRC and reveals that disrupting the CLDN1/CD81 axis might represent a novel treatment strategy.

## 1 Introduction

Globally, colorectal carcinoma (CRC) is the third most commonly diagnosed cancer and the second leading cause of cancer-related death [1]. Its increasing prevalence is attributable to a combination of demographic factors (age and sex), inflammatory bowel disease, and changes in lifestyle and environment [2]. More than 90% of CRC mortality is caused by metastasis, with a five-year survival rate of <15% for patients diagnosed at this advanced stage [3]. The metastasis of CRC is a multifactorial process governed by numerous molecular mechanisms. Studies have demonstrated that epithelial–mesenchymal transition (EMT) enhances the invasive and migratory properties of CRC cells, facilitating the progression from local tissue invasion to distant organ colonization [4]. An immunosuppressive tumor microenvironment not only suppresses immune responses but also promotes metastasis by secreting a specialized, pro-regenerative extracellular matrix [5]. Therefore, a deeper understanding of the mechanisms driving CRC metastasis is essential for improving the prevention and treatment of metastatic CRC.

Accumulating evidence underscores the critical role of cell membrane proteins in orchestrating metastatic behaviors and tumor progression [6,7]. However, the precise underlying molecular mechanisms remain less understood. As a key component of tight junctions, claudin-1 (CLDN1) is predominantly expressed in epithelial cell membranes, where it governs the formation and maintenance of intercellular junctions [8]. Recent evidence has established a close link between CLDN1 and tumor progression, as well as metastasis. CLDN1 facilitates the progression of head and neck squamous cell carcinoma by regulating EMT via the adenosine 5′-monophosphate-activated protein kinase (AMPK)/transforming growth factor-beta (TGF-β) signaling pathway [9]. CLDN1 inhibits apoptosis and facilitates metastasis and growth of esophageal squamous cell carcinoma via the AMPK/signal transducer and activator of transcription 1 (STAT1)/Unc-51-like autophagy activating kinase 1 (ULK1) and phosphoinositide 3-kinase-protein kinase B-mammalian target of rapamycin (PI3K-AKT-mTOR) signaling pathways [10,11]. Notably, CLDN1 promotes EMT and CRC metastasis via the AMPK signaling pathway [8]. A recent study revealed that dysregulation of CLDN1 in CRC induces aberrant protein‒protein interactions, promoting cancer stemness and chemoresistance [12].

CD81 is a tetraspanin membrane protein that interacts with diverse partners to form extensive molecular networks, supporting its multiple biological functions. Interestingly, the role of CD81 markedly varies across different cancer types. It facilitates the cell invasion, migration, and proliferation of melanoma and osteosarcoma via the AKT and extracellular signal-regulated kinase (ERK) signaling pathway [13,14]. Conversely, CLDN1 exerts an inhibitory effect on bladder cancer cell invasion, an action mediated by the ERK signaling pathway [15]. CD81 plays a suppressive role in gastric cancer tumorigenesis by inhibiting p38 signaling [16]. It also inhibits the proliferation of hepatocellular carcinoma cells via the c-Jun N-terminal kinase (JNK) signaling pathway [17]. However, the effects of CD81 on CRC progression remain unclear.

The present study was designed to outline the regulatory role of CLDN1 in CRC cell tumorigenicity through its interaction with CD81, elucidating the underlying signaling cascade.

## 2 Materials and Methods

### 2.1 Bioinformatics Analysis

The Cancer Genome Atlas (TCGA)-Colon Adenocarcinoma (COAD) dataset (https://ualcan.path.uab.edu/analysis.html) provided gene expression profiles for 286 tumor tissues and 41 normal tissues, which were used for analysis. The association between overall survival and CLDN1 expression in colon cancer was analyzed using the Kaplan‒Meier plotter database [18]. The Search Tool for Recurring Instances of Neighboring Genes (STRING) database (version 12.0, https://cn.string-db.org/) was employed to construct protein-protein interaction networks, with Cytoscape software (version 3.10.1, Cytoscape Team, San Diego, CA, USA) used for network visualization [19].

### 2.2 Tissue Microarray

The CRC tissue microarray (Shanghai Zhuoli Biotech Company, ZL-Cocsur1241, Shanghai, China) comprised 69 tumor and 55 adjacent normal tissue samples. The clinical features of patients with CRC are listed in Table 1. This study was approved by the Ethics Committee of the Shanghai Zhuoli Biotech Company (approval number IRBS-Cocsur1241-25-010). Written informed consent was obtained from all participants prior to enrollment, and all study procedures were performed in strict adherence to the principles of the Declaration of Helsinki.

**Table 1:** Clinical features of patients with colorectal cancer (CRC).

Parameters	Number of Patients (N = 69)
**Age, n (%)**	
<70	33 (47.8%)
≥70	36 (52.2%)
**Gender, n (%)**	
Female	35 (50.7%)
Male	34 (49.3%)
**Pathological TNM stage, n (%)**	
I	4 (5.8%)
II	41 (59.4%)
III	12 (17.4%)
IV	12 (17.4%)
**Pathological T stage, n (%)**	
T1	5 (7.3%)
T2	19 (27.5%)
T3	37 (53.6%)
T4, n (%)	8 (11.6%)
**Pathological N stage, n (%)**	
N0	46 (66.7%)
N1	16 (23.2%)
N2	7 (10.1%)
**Pathological M stage, n (%)**	
M0	57 (82.6%)
M1	12 (17.4%)
**Grade, n (%)**	
G2	62 (89.9%)
G3	7 (10.1%)

### 2.3 Immunohistochemistry (IHC)

IHC was performed on a CRC tissue microarray to evaluate the expression of CLDN1 and CD81 proteins in CRC and their prognostic importance. The tissue section was cultured with 3% hydrogen peroxide in deionized water for 30 min to inactivate endogenous peroxidase activity, followed by blocking with goat serum (Servicebio Technology Co., Ltd., G1208-5ML, Wuhan, China) at 25°C for 15 min. The section was then incubated overnight at 4°C with primary antibodies against CLDN1 (Proteintech, 28674-1-AP, 1:1000, Rosemont, IL, USA) and CD81 (Proteintech, 27855-1-AP, 1:1000), followed by incubation for 30 min at 25°C with a secondary antibody (Cell Signaling Technology, 8114, 1:400, Danvers, MA, USA) conjugated to horseradish peroxidase, and visualized with 3,3′-diaminobenzidine (Beyotime Biotechnology, ST3205-5 g, Shanghai, China) for 10 min at 25°C. Two blinded pathologists independently quantified the immunostaining results via a method incorporating two components: (1) histopathological evaluation, which employs a five-tiered scoring system (0–4; 0 for <5%, 1 for 5%–25%, 2 for 25%–50%, 3 for 50%–75%, and 4 for >75% positivity) to quantify the extent of cellular staining, and (2) examination of the staining intensity, evaluated using a scale ranging from 0 to 3 (0 for negative, 1 for weak, 2 for moderate, and 3 for strong). The composite score (range: 0–12) was calculated by multiplying these two values. Using a predetermined score cutoff of 6, the samples were dichotomized into high and low CLDN1 expression groups, and survival outcomes were compared between the two subgroups.

### 2.4 Cell Culture and Treatment

Human CRC cell lines SW620 (CCL-227), HCT116 (CCL-247), HT29 (HTB-38), normal intestinal epithelial cells HIEC-6 (CRL-3266), and 293T cells (CRL-3216) were purchased from the American Type Culture Collection (ATCC, Manassas, VA, USA). The cells were authenticated by short tandem repeat profiling, confirmed to be mycoplasma-free and cultured in medium supplemented with 10% heat-inactivated fetal bovine serum (FBS; Corning Inc., 35-081-CV, Corning, NY, USA) and 1% antibiotic-antimycotic solution (Solarbio Science & Technology Co., Ltd., P1400-100, Beijing, China) under standard conditions (37°C, 5% CO_2_). Specifically, HT29 and SW620 cells were maintained in Roswell Park Memorial Institute (RPMI) 1640 Medium (Labgic Technology Co., Ltd., 11875-093, Beijing, China), and Dulbecco’s Modified Eagle’s Medium (DMEM, Labgic Technology Co., Ltd., BL301A) was used for culturing HCT116 cells. SW620 cells were treated with 1 μg/mL of anti-CD81 monoclonal antibody (mAb) 5A6 (Sigma‒Aldrich, MABF2061, St. Louis, MO, USA) or isotype control mAb (Sigma‒Aldrich, I5154) for 48 h, as described previously [20].

### 2.5 Gene Overexpression and Knockdown

Three independent short hairpin RNA (shRNA) sequence designed to target CLDN1 (shCLDN1-1, 5′-GAATCGTTCAAGAATTCTATG-3′; shCLDN1-2, 5′-AGTGGAGGATTTACTCCTATG-3′; shCLDN1-3, 5′-CAAGAATTCTATGACCCTATG-3′) or CD81 (shCD81-1, 5′-CCTCAGTGCTCAAGAACAATT-3′; shCD81-2, 5′-GGATGTGAAGCAGTTCTATG-3′; shCD81-3, 5′-GCACCAAGTGCATCAAGTACC-3′) was engineered into the pLKO.1 puro lentiviral vector (Addgene, 8453, Watertown, MA, USA). Lentivirus was produced by cotransfecting 293T cells with shRNA constructs, psPAX2 (Addgene, 12260), and pMD2. G (Addgene, 12259) plasmid using Lipofectamine 2000 (Invitrogen, 11668500, Carlsbad, CA, USA). Recombinant lentivirus harvested 48 h posttransfection were subsequently used to infect HT29 and HCT116 cells. HT29 and HCT116 cells were seeded in 6-well plates at a density of 5 × 10^5^ cells per well and single transduced with the recombinant lentivirus at a multiplicity of infection of 20, supplemented with 8 μg/mL polybrene (Sigma‒Aldrich, TR-1003), at 37°C for one day. Stable transduct was then established by 3 μg/mL puromycin (Gibco, A1113803, Grand Island, NY, USA) selection for 4 more days. Parallel overexpression studies employed pcDNA3.1(+) vector (Addgene, V790-20) harboring the full-length coding sequence of CLDN1 or CD81, which was used for transfecting SW620 cells using Lipofectamine 2000. The appropriate controls included nontargeting shRNA (shNC) in pLKO.1 puro and empty pcDNA3.1(+) vector transfections.

### 2.6 Cell Viability Assay

For cell viability assessments, cells were plated in 96-well culture plates at a density of 5 × 10^3^ cells/well at 37°C in a humidified incubator with 5% CO_2_. Following 12, 24, or 48 h incubation, 10 μL of Cell Counting Kit-8 (CCK-8) reagent (Beyotime Biotechnology, C0039) was added to each well. The optical density was determined using a Multiskan FC microplate reader (Thermo Fisher Scientific, Waltham, MA, USA) at 450 nm after 60 min of enzymatic reaction. The blank wells (medium plus CCK-8 without cells) were included for background subtraction. Three independent biological experiments were performed, along with three technical replicates per condition.

### 2.7 Wound Healing Assay

A scratch (wound healing) assay was used to evaluate cell migration as described previously [3]. Following the experimental interventions, CRC cells were plated in 6-well culture dishes at a density of 5 × 10^5^ cells/well and grown until 100% confluency was achieved. Mechanical wounds were generated using sterile 200 μL pipette tips under microscopy (Leica DM3000, Heidelberg, Germany). Phosphate-buffered saline was used to wash cells to remove cellular debris, and cells were then placed back in the incubator for culturing in serum-free RPMI1640 medium (Labgic Technology Co., Ltd., 11875-093) or DMEM (Labgic Technology Co., Ltd., BL301A). Time-lapse images of wounds at premarked reference points were obtained at 0, 24, and 48 h postwounding, and the scratch width was quantified using ImageJ software (National Institutes of Health, Bethesda, MD, USA). The wound closure rates were calculated using the formula [(wound width at 0 h–width at 24 h or 48 h)/width at 0 h] × 100%. Three independent biological experiments were performed, along with three technical replicates per condition.

### 2.8 Transwell Invasion Assay

Cell invasive capacity was assessed using Matrigel (Corning Inc., 356234)-coated Transwell chambers (8 μm, Corning Inc., 3464) as described previously [3]. Briefly, Matrigel was diluted to 1 mg/mL with serum-free medium, and 100 μL of the diluted solution was added to upper chamber of Transwell insert for 1 h at 37°C. CRC cells (1.5 × 10^4^/well) were seeded in the upper chamber, while the lower chamber contained 700 μL of culture medium supplemented with 10% FBS as the chemoattractant. After 48 h of incubation, noninvading cells were removed, and the migrated cells on the underside of the membrane were fixed with 4% paraformaldehyde for 10 min and stained with 0.5% crystal violet (Solarbio Science & Technology Co., Ltd., 0528) for 20 min. Image of randomly selected microscopic field was captured using a XDS-500C microscope (Caikon optical instrument Co., Ltd., Shanghai, China). Three independent biological experiments were performed, along with three technical replicates per condition.

### 2.9 Quantitative Real-Time Polymerase Chain Reaction (qRT‒PCR)

RNA was isolated from CRC cell lines using TRIzol Reagent (Invitrogen, 15596018CN), followed by DNase I (2 μL, 10 U; Takara Biomedical Technology Co., Ltd., 2270A, Beijing, China) treatment for 30 min at 37°C to eliminate genomic DNA contamination. The cDNA Synthesis Kit (Yeasen Biotechnology Co., Ltd., 11119ES60, Shanghai, China) with 100 ng of total RNA per reaction was used to generate First-strand cDNA, according to the manufacturer’s specifications. The reaction mixture (20 μL) contained 5 × Hifair^®^ II Buffer (4 μL), Hifair^®^ II Enzyme Mix (2 μL), Oligo (dT)_18_ (50 μM, 1 μL; Thermo Fisher Scientific, SO132), total RNA (1 μL), and RNase-free H_2_O (12 μL). The PCR was carried out as following condition: 25°C for 5 min, 42°C for 30 min, and 85°C for 5 min. qRT‒PCR was carried out on an Applied Biosystems 7500 Real-Time PCR System (Applied Biosystems, Thermo Fisher Scientific) using Hieff^®^ qPCR SYBR Green Master Mix (Yeasen Biotechnology Co., Ltd., 11203ES03) with 20 ng cDNA per reaction. qPCR was carried out in a 20-μL reaction mixture with SYBR Green Master Mix (10 μL), forward primers (0.2 μM, 0.4 μL), reverse primers (0.2 μM, 0.4 μL), cDNA template (1 μL), and diethylpyrocarbonate-treated H_2_O (8.2 μL). The PCR cycling conditions were as follows: initial denaturation at 95°C for 10 min; 40 cycles of 95°C for 15 s and 60°C for 45 s; and a final melt curve stage consisting of 95°C for 15 s, 60°C for 1 min, 95°C for 15 s, and 60°C for 15 s. The relative gene expression was quantified using the comparative 2^−∆∆Ct^ method and normalized to endogenous glyceraldehyde-3-phosphate dehydrogenase (GAPDH) expression. The following primer sequences were employed for qPCR analysis: CLDN1-F, 5′-ATGACCCCAGTCAATGCCAG-3′; CLDN1-R, 5′-GCTGGAAGGTGCAGGTTTTG-3′; CD81-F, 5′-CCTGGTCATCCTGTTTGCCT-3′; CD81-R, 5′-TGCTTCACATCCTTGGCGAT-3′; GAPDH-F, 5′-GTCAAGGCTGAGAACGGGAA-3′; and GAPDH-R, 5′-AAATGAGCCCCAGCCTTCTC-3′. Three independent biological experiments were performed, along with three technical replicates per condition.

### 2.10 Western Blot Analysis

Cellular protein was extracted with radioimmunoprecipitation assay lysis buffer (Thermo Fisher Scientific, 89901), and protein concentrations were determined using a bicinchoninic acid protein detection kit (Sigma‒Aldrich, BCA1-1KT). Subsequently, the protein lysates (30 μg) were separated on 10% or 12% sodium dodecyl sulfate‒polyacrylamide gel electrophoresis (SDS‒PAGE) and electrotransferred to nitrocellulose membrane. After blocking with 5% nonfat milk at 25°C for 1 h, the membrane was incubated with specific primary antibody against CLDN1 (Cell Signaling Technology, 13255, 1:1000), CD81 (Cell Signaling Technology, 56039, 1:1000), and GAPDH (Proteintech, 60004-1-Ig, 1:5000) overnight at 4°C. Membrane was then probed with the corresponding horseradish peroxidase-conjugated goat anti-rabbit (ZSGB-BIO, ZB-2301, 1:5000, Beijing, China) or goat anti-mouse (ZSGB-BIO, ZB-2305, 1:5000) secondary antibody at 25°C for 1 h. Protein bands were visualized using Clarity Electrochemiluminescence Substrate (Bio-Rad, 1705060, Hercules, CA, USA). Three independent biological experiments were performed, along with three technical replicates per condition.

### 2.11 Co-IP and Ubiquitination Analyses

For Co-IP analysis of the CLDN1-CD81 interaction, the cells were lysed in ice-cold Co-IP buffer for 20 min. The lysates were centrifuged at 10,000× *g* for 10 min at 4°C, and supernatants (200 μL) were divided into three groups: input, IgG control, and IP. In the input group, 2.5 μg of protein was used per immunoprecipitation reaction. The IgG control group received 2 μL of isotype control IgG (Santa Cruz Biotechnology, sc-515946, 1:100, Santa Cruz, CA, USA), while the IP group was incubated with 2 μL of anti-CLDN1 antibody (Cell Signaling Technology, 13255, 1:200) or 2 μL of anti-CD81 antibody (Cell Signaling Technology, 10037, 1:50) overnight at 4°C. Subsequently, protein complexes were captured by incubation with 40 μL of protein A/G magnetic beads (Thermo Fisher Scientific, 88803) with gentle rotation at 4°C for 12 h. The immunocomplex bound to the beads was then collected by centrifugation at 4°C, followed by washing with IP buffer (20 mM Tris, 150 mM NaCl) for four times to remove nonspecific binding, and the bound proteins were eluted in 20 μL of 2× SDS‒PAGE loading buffer for 5 min at 95°C. The immunoprecipitated proteins (25 μg) in the IgG control and IP groups and proteins (20 μg) in the input group were resolved via 10% SDS‒PAGE and immunoblotted with anti-CLDN1 (Cell Signaling Technology, 13255, 1:1000), anti-CD81 (Cell Signaling Technology, 56039, 1:1000), or anti-ubiquitin (Abcam, ab134953, 1:2000, Waltham, MA, USA) antibodies.

### 2.12 Immunofluorescence

Immunofluorescence staining was conducted on fixed and permeabilized HT29 cells. HT29 cells were fixed with 4% paraformaldehyde for 15 min, permeabilized with Triton X-100 for 20 min at 25°C, followed by blocking with bovine serum albumin (Sigma‒Aldrich, V900933) for 1 h. The cells were then incubated with anti-CLDN1 (Proteintech, 28674-1-AP, 1:500) or anti-CD81 (Proteintech, 27855-1-AP, 1:500) antibodies overnight at 4°C, followed by Alexa Fluor 555-labeled goat anti-rabbit IgG (Beyotime Biotechnology, A0453, 1:200) or Alexa Fluor 488-labeled goat anti-rabbit IgG (Beyotime Biotechnology, A0423, 1:200) for 30 min at 25°C. Cell nucleus was counterstained with 4′,6-diamidino-2-phenylindole (1 μg/mL, Beyotime Biotechnology, C1002) for 15 min, and image was captured using an Olympus BX41 phase contrast microscope (Olympus Corporation, Shinjuku-ku, Japan). Three independent biological experiments were performed, along with three technical replicates per condition.

### 2.13 Tumor Xenograft

Twenty-four BALB/c nude mice (male, aged 6 weeks, weighted 18–22 g; Ziyuan Experimental Animal Technology Co., Ltd., Hangzhou, China) were housed in a specific pathogen-free-grade equipped animal facility with 20–25°C and 50 ± 5% humidity, following a consistent 12-h dark/12-h light cycle, with free access to autoclaved food and water. After a 7-day acclimatization period in the animal facility, nude mice were randomly assigned to four groups with different treatments, including control group, CLDN1+5A6 group, CLDN1+isotype mAb group, and Vector+isotype mAb group. In control group, SW620 cells (5 × 10^6^) were orthotopically engrafted into the left flank of BALB/c nude mice. In the CLDN1+5A6 group, on day 12, CLDN1-overexpressing SW620 cells (5 × 10^6^)-bearing BALB/c nude mice were injected with 100 μg of anti-CD81 mAb 5A6 intraperitoneally and weekly thereafter for three weeks as described previously [20,21]. In CLDN1+isotype mAb, on day 12, CLDN1-overexpressing SW620 cells (5 × 10^6^)-bearing BALB/c nude mice were injected with 100 μg of isotype control mAb as mentioned above. In the Vector+isotype mAb group, on day 12, blank vector-expressing SW620 cells (5 × 10^6^)-bearing BALB/c nude mice were injected with 100 μg of isotype control mAb as mentioned above. Tumor volume was monitored every three days and calculated using the formula: (length × width^2^)/2. The animals were euthanized by exposure to 2% isoflurane (Sigma‒Aldrich, 792632) anesthesia for 3 min, followed by 40% CO_2_ for 5 min on days 33 or earlier if predefined humane endpoints (tumor diameter exceeding 2 cm or body weight loss exceeding 20%). Then, the tumors were harvested (n = 6 mice per group). No animals were excluded from the experiment. All animal experiments were conducted in accordance with the National Research Council’s Guide for the Care and Use of Laboratory Animals and followed the ARRIVE guidelines. Approval for the animal research was obtained from the Ethics Committee of Shanghai Rat & Mouse Biotech Ltd. (Approval no. RM202402(33)).

### 2.14 Statistical Analysis

Experiments were carried out using triplicate independent biological replicates. Quantitative data are presented as the means ± standard deviations. Statistical analysis was carried out using GraphPad Prism 8.4.2 (GraphPad Software, San Diego, CA, USA). Comparison between two groups was made using Student’s *t* test. Statistical significance among multiple groups was determined using one-way ANOVA with Dunnett’s post hoc test, and *p* < 0.05 was considered statistically significant.

## 3 Results

### 3.1 CLDN1 Expression Is Increased in Colon Cancer Tissue and CRC Cell Line

**Figure 1:**
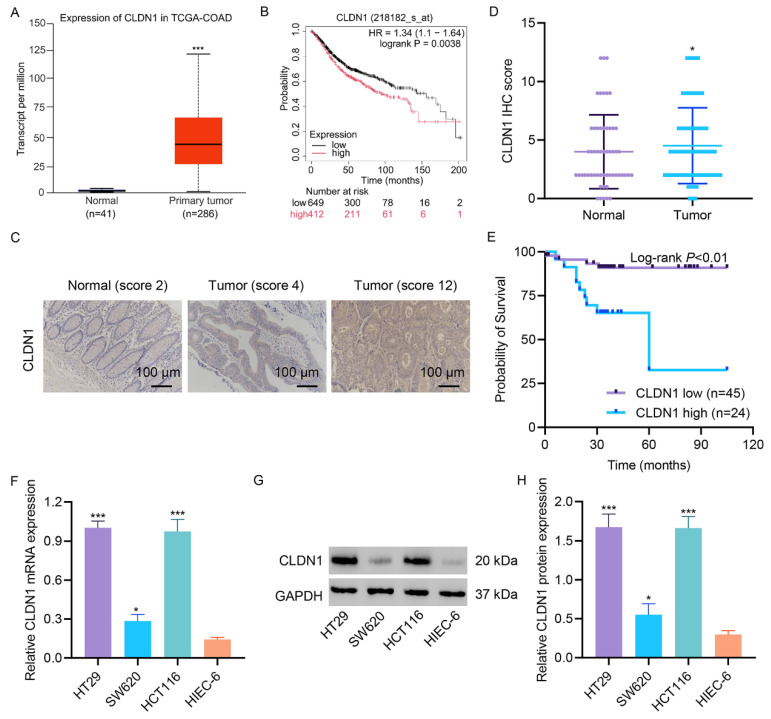
**Claudin-1 (CLDN1) expression is increased in colon cancer tissue and CRC cell line.** (**A**) CLDN1 expression levels in the TCGA-COAD database. (**B**) Overall survival of CRC patients stratified by CLDN1 expression using the Kaplan‒Meier plotter database. (**C**,**D**) Expression levels of CLDN1 in normal and tumor tissues in CRC tissue microarrays. (**E**) Survival analysis of CLDN1 expression in CRC tissue microarrays. (**F**–**H**) Expression levels of CLDN1 in CRC cell lines and normal intestinal epithelial cells (HIEC-6). Scale bar, 100 μm. *p < 0.05, ***p < 0.001 vs. normal or HIEC-6 cells.

Analysis of gene expression profiles from the TCGA database revealed significant upregulation of CLDN1 expression in the colon cancer tissues compared to normal colon tissues (Fig. 1A). Consistently, survival analysis of CRC patients in the Kaplan‒Meier plotter database reveale d that high CLDN1 expression correlated with markedly poorer overall survival (Fig. 1B). CRC tissue microarrays further confirmed that CLDN1 protein expression was markedly increased in colon cancer tissue compared to normal colon tissue (Fig. 1C,D). CLDN1 expression emerged as a significant prognostic factor, with high expression levels associated with markedly reduced patient survival (Fig. 1E). Additionally, evaluation of CLDN1 expression across CRC cell lines revealed pronounced upregulation at the mRNA and protein levels compared to normal intestinal epithelial cells (HIEC-6), underscoring its tumor-specific overexpression (Fig. 1F–H).

### 3.2 CLDN1 Knockdown Suppresses CRC Cell Viability, Migration, and Invasion

**Figure 2:**
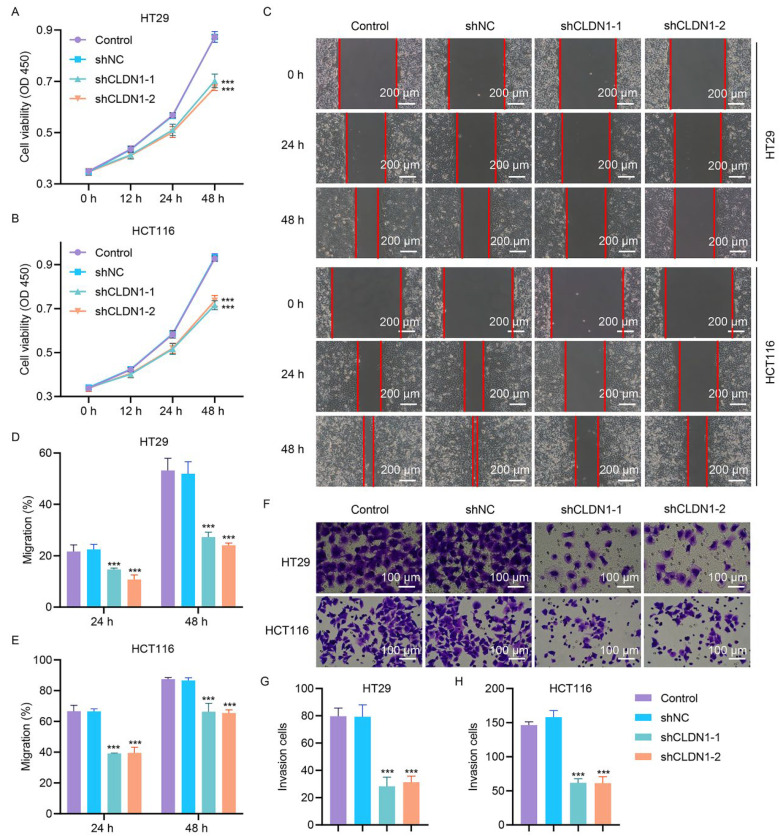
**CLDN1 silencing inhibits the viability, migration, and invasion of CRC cells.** Following lentiviral transduction with CLDN1 shRNA or shNC in HT29 and HCT116 cells, (**A**,**B**) cell viability, (**C**–**E**) migration, and (**F**–**H**) invasion were detected via CCK-8, scratch wound healing and Transwell assay. C, Scale bar, 200 μm. F, Scale bar, 100 μm. ***p < 0.001 vs. shNC.

Based on their high endogenous CLDN1 expression, HT29 and HCT116 cell lines with higher CLDN1 expression levels were transduced with shRNAs (shCLDN1-1, -2, and -3) to establish stable knockdown models. There were no significant differences in the knockdown efficiency between shCLDN1-1, shCLDN1-2, and shCLDN1-3; thus, cells randomly transduced with shCLDN1-1 and shCLDN1-2 were subsequently used to examine the effects of CLDN1 loss on cell viability, invasion, and migration (Fig. S1A‒C). The CCK-8 assay showed that CLDN1 depletion markedly suppressed the viability of HT29 and HCT116 cells (Fig. 2A,B). Wound-healing assays revealed that CLDN1 knockdown significantly attenuated cell migration 24 and 48 h posttransfection (Fig. 2C–E). Furthermore, Matrigel-based invasion assays showed that CLDN1 knockdown robustly reduced the invasion of CRC cells 48 h posttransfection (Fig. 2F–H).

### 3.3 CLDN1 Interacts with CD81 and Stabilizes Its Expression via Ubiquitination Regulation

**Figure 3:**
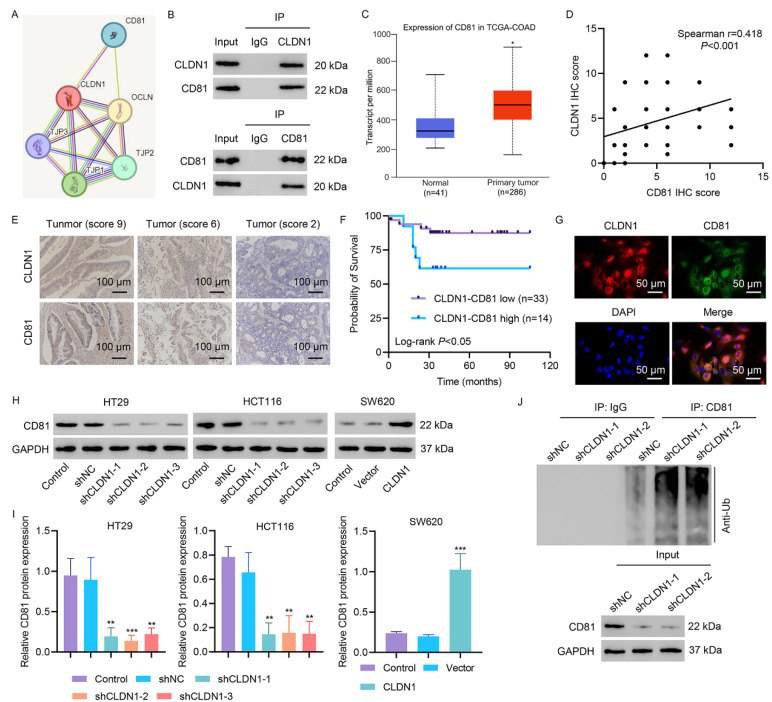
**CLDN1 binds to CD81 and enhances its expression by suppressing ubiquitination.** (**A**) Cytoscape-predicted CLDN1 interactome. (**B**) Co-IP verification of the CLDN1-CD81 interaction. (**C**) TCGA-based CD81 expression in normal and colon cancer tissues. (**D**,**E**) Tissue microarray analysis of the CLDN1-CD81 correlation. (**F**) Survival analysis of CLDN1-CD81 coexpression in CRC tissue microarrays. (**G**) Immunofluorescence staining confirmed the colocalization of CLDN1 and CD81 in HT29 cells. (**H**,**I**) Reduced CD81 in CLDN1-silenced HT29/HCT116 cells and increased CD81 in CLDN1-overexpressing SW620 cells. (**J**) CD81 ubiquitination in CLDN1-knockdown HT29 cells. E, Scale bar, 100 μm. G, Scale bar, 50 μm. *p < 0.05, **p < 0.01, ***p < 0.001 vs. normal, shNC, or vector.

Protein‒protein interaction network was built using the STRING database, and protein interactions were analyzed. CD81 was found to interact with CLDN1 (Fig. 3A). To establish CD81 as a functional effector downstream of CLDN1, we first investigated its physical interaction with CLDN1. Co-IP assays revealed that CLDN1 and CD81 reciprocally bound in CRC cells, as both proteins were strongly detected in their respective immunoprecipitates (Fig. 3B). Analysis of the TCGA-COAD dataset revealed significant upregulation of CD81 in tumor tissues compared to normal tissues (Fig. 3C). IHC staining of CRC tissues confirmed the coexpression of CLDN1 and CD81, with their protein levels strongly positively correlated (Fig. 3D,E). To examine the relationship between CLDN1-CD81 coexpression levels and clinical parameters, we categorized the CRC patients into two subgroups: the CLDN1-CD81 low group (patients with low expression of CLDN1 and CD81) and the CLDN1-CD81 high group (patients with high expression of CLDN1 and CD81). As shown in Table 2, high coexpression of CLDN1 and CD81 was associated with advanced tumor grades and pathological TNM stages, suggesting their important roles in tumor growth and metastasis. Moreover, patients with high CLDN1-CD81 coexpression exhibited a lower survival rate (Fig. 3F). Immunofluorescence staining confirmed the colocalization of CLDN1 and CD81 in HT29 cells (Fig. 3G). CLDN1 silencing significantly reduced CD81 protein levels in HT29 and HCT116 cells, whereas CLDN1 overexpression in SW620 cells markedly upregulated CD81 expression (Fig. 3H,I). To elucidate the mechanistic basis of this regulation, we assessed the impact of CLDN1 on CD81 ubiquitination. CLDN1 knockdown substantially enhanced CD81 ubiquitination (Fig. 3J). These findings collectively demonstrate that CLDN1 stabilizes CD81 by attenuating its ubiquitination, thereby maintaining oncogenic CD81 levels in CRC cells.

**Table 2:** Relationship between claudin-1 (CLDN1)-CD81 coexpression and clinical features of patients with CRC.

	Low (N = 33)	High (N = 14)	*p* Value
**Age, years, mean (SD)**	69.5 (12.1)	70.0 (13.0)	0.902
**Gender, n (%)**			0.321
Female	17 (51.5%)	5 (35.7%)	
Male	16 (48.5%)	9 (64.3%)	
**Pathological TNM stage, n (%)**			0.042
I	4 (12.1%)	0 (0.0%)	
II	22 (66.7%)	7 (50.0%)	
III	4 (12.1%)	1 (7.1%)	
IV	3 (9.1%)	6 (42.9%)	
**Pathological T stage, n (%)**			0.012
T1	4 (12.1%)	0 (0.0%)	
T2	15 (45.4%)	3 (21.4%)	
T3	12 (36.4%)	5 (35.7%)	
T4	2 (6.1%)	6 (42.9%)	
**Pathological N stage, n (%)**			0.027
N0	26 (78.8%)	7 (50.0%)	
N1	6 (18.2%)	3 (21.4%)	
N2	1 (3.0%)	4 (28.6%)	
**Pathological M stage, n (%)**			0.007
M0	30 (90.9%)	8 (57.1%)	
M1	3 (9.1%)	6 (42.9%)	
**Grade, n (%)**			0.034
G2	31 (93.9%)	10 (71.4%)	
G3	2 (6.1%)	4 (28.6%)	

Differences between groups were determined by the chi-square test.

### 3.4 CD81 Knockdown Impairs CRC Cell Viability, Migration, and Invasion

**Figure 4:**
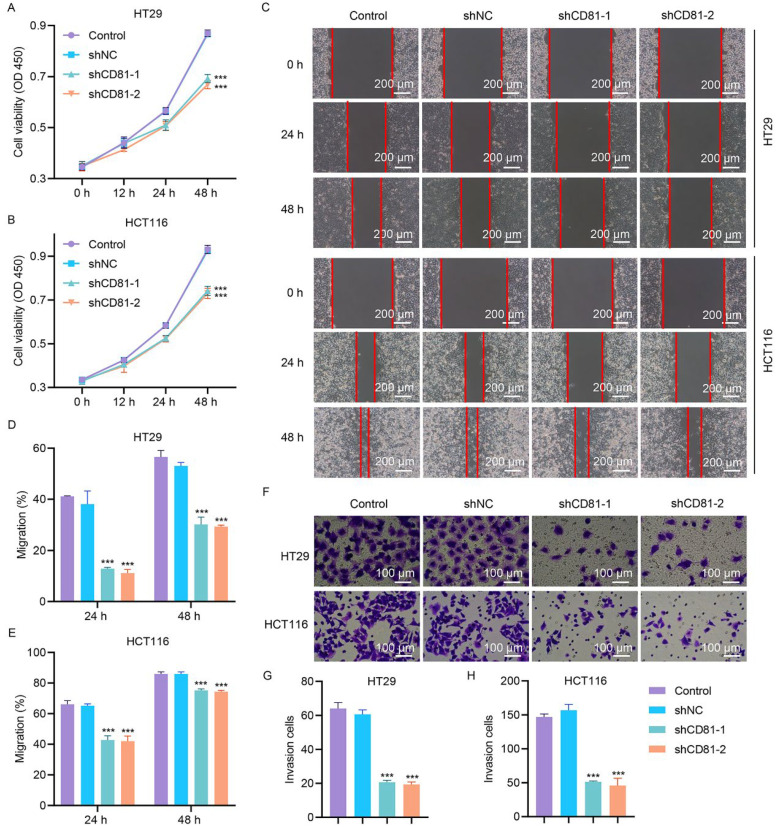
**CD81 silencing attenuates the metastatic ability and viability of CRC cells.** In CD81-knockdown HT29/HCT116 cells, (**A**,**B**) cell viability, (**C**–**E**) migration, and (**F**–**H**) invasion were measured via CCK-8, scratch wound healing and Transwell assay. C, Scale bar, 200 μm. F, Scale bar, 100 μm. ***p < 0.001 vs. shNC.

The knockdown efficiency of CD81 in HT29 cells transduced with the CD81 shRNA lentiviral vector was validated, and substantial reductions in the mRNA and protein levels were detected (Fig. S1D–F). There were no significant differences in the knockdown efficiency between shCD81-1, shCD81-2, and shCD81-3. To examine the consequences of CD81 ablation in CRC progression, HT29 and HCT116 cells were randomly transduced with shCD81-1 and shCD81-2. The CCK-8 assay showed that silencing of CD81 ablation markedly reduced the viability of HT29 and HCT116 cells (Fig. 4A,B). Wound-healing assays revealed a pronounced decrease in the migratory capacity of CD81-deficient cells compared to the control cells (Fig. 4C–E). Consistently, Matrigel-based transwell assays revealed that CD81 silencing robustly attenuated the invasive potential of CRC cells (Fig. 4F–H).

### 3.5 Functional Validation of the CLDN1/CD81 Oncogenic Axis In Vitro and In Vivo

**Figure 5:**
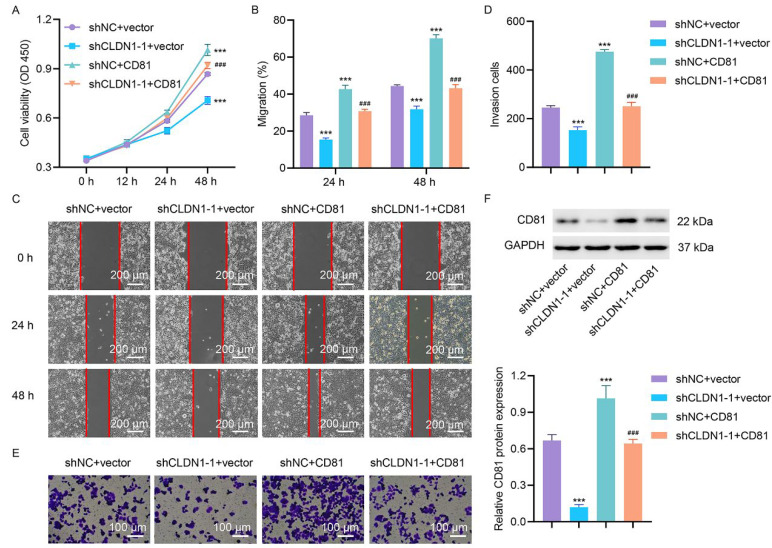
**CD81 overexpression reverses CLDN1 silencing-mediated reductions in CRC cell viability, migration, and invasion.** After transducing HT29 cells with CLDN1 shRNA or shNC and transfecting them with CD81 expression or a blank vector, (**A**) cell viability, (**B**,**C**) migration, (**D**,**E**) invasion, and (**F**) expression levels of CD81 were detected via CCK-8, scratch wound healing, Transwell assay and Western blot. C, Scale bar, 200 μm. E, Scale bar, 100 μm. ***p < 0.001 vs. shNC + vector. ^###^*p* < 0.001 vs. shCLDN1-1 + vector.

To investigate the role of CD81 in CLDN1-mediated CRC progression, we transduced HT29 cells with either shCLDN1-1 or shNC, along with CD81 expression or a blank vector. CD81 overexpression reversed the functions of CLDN1 silencing in the viability, migratory, and invasive capabilities of HT29 cells (Fig. 5A‒E). Additionally, CD81 overexpression reversed the CLDN1-mediated decrease in CD81 protein expression (Fig. 5F).

**Figure 6:**
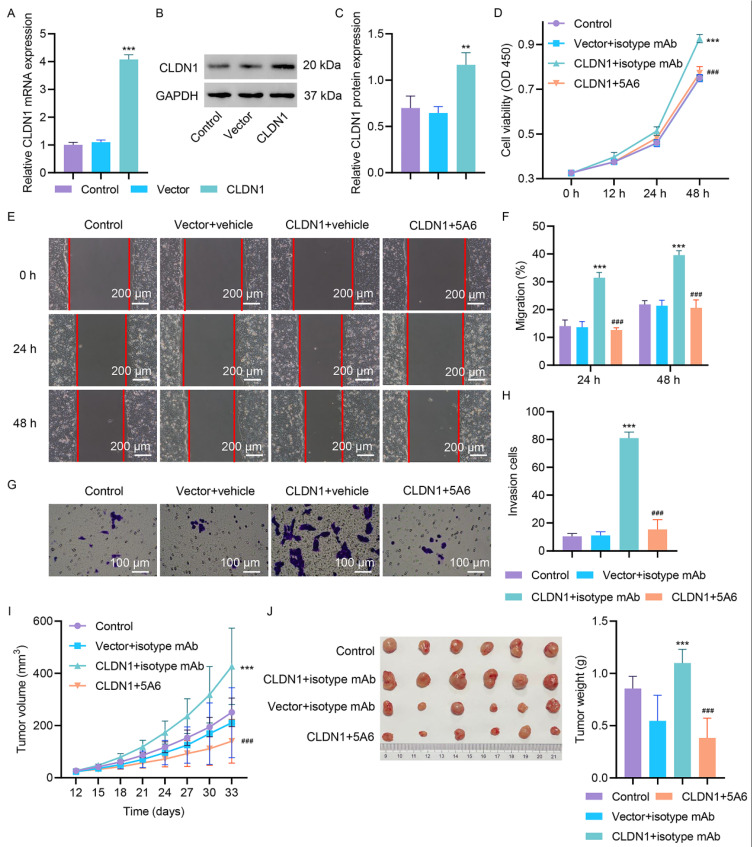
**The anti-CD81 mAb 5A6 reverses CLDN1 overexpression-mediated oncogenicity in SW620 cells and tumor xenografts.** (**A**–**C**) CLDN1-overexpressing SW620 cell validation by qRT‒PCR and Western blot. SW620 cells with either CLDN1 expression or a blank vector transfection were treated with the anti-CD81 mAb 5A6 or an isotype control mAb, and (**D**) cell viability, (**E**,**F**) migration, and (**G**,**H**) invasion were detected via CCK-8, scratch wound healing and Transwell assay. (**I**) Tumor volume and (**J**) tumor weight of mice injected with SW620 cells transfected with either CLDN1 expression or a blank vector, alone or in combination with the anti-CD81 mAb 5A6 or the isotype control mAb. E, Scale bar, 200 μm. G, Scale bar, 100 μm. **p < 0.01, ***p < 0.001 vs. vector or vector + isotype mAb. ^###^*p* < 0.001 vs. CLDN1 + isotype mAb.

We treated CLDN1-overexpressing SW620 cells with the anti-CD81 mAb 5A6 to investigate the functional dependence of CLDN1-driven oncogenicity on its interaction with CD81. CLDN1 was overexpressed in SW620 cells through plasmid transfection, and its successful overexpression was confirmed by qRT‒PCR and Western blot (Fig. 6A‒C). Cotreatment with 5A6 countered the protumorigenic effects mediated by CLDN1. Cell viability, migration capacity, and invasive potential were significantly lower in the 5A6 + CLDN1-overexpressing group than in the CLDN1-overexpressing group (Fig. 6D–H). CLDN1-overexpressing SW620 cells were subcutaneously injected into nude mice to establish xenograft models. On day 12, mice were intraperitoneally injected with 100 μg of 5A6 or an isotype control mAb. CLDN1 overexpression increased tumor volume (Fig. 6I) and weight (Fig. 6J). These effects were inhibited by 5A6, indicating that the CLDN1/CD81 axis might regulate tumor growth *in vivo*.

## 4 Discussion

### 4.1 Interpretation

**Figure 7:**
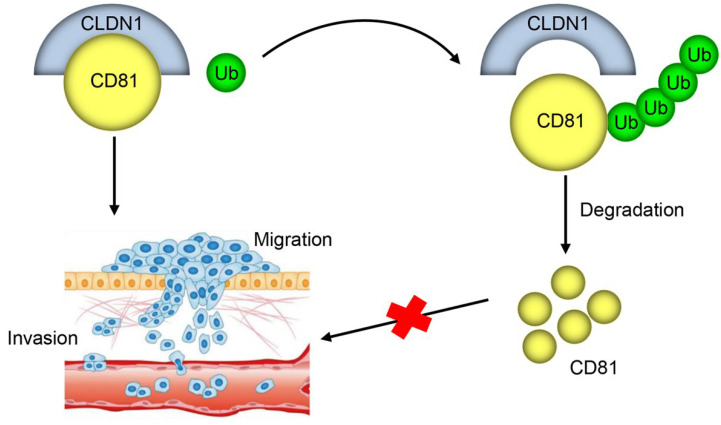
**Molecular mechanisms of CLDN1-CD81 regulation in CRC.** CLDN1 promotes cell migration and invasion of CRC by binding to CD81 and suppressing its ubiquitination, thereby stabilizing CD81 expression.

Machine learning has identified CLDN1 as a key biomarker with significant diagnostic potential in CRC [22]. The current study provides compelling evidence that CLDN1 promotes CRC progression by binding to CD81 and suppressing its ubiquitination, thereby stabilizing CD81 expression (Fig. 7). This novel regulatory mechanism offers important insights into how membrane protein interactions can influence oncogenic signaling pathways in CRC.

A previous study suggested that the expression of different CLDN family genes is closely associated with pathological stage and immune cell infiltration in CRC [20]. CLDN family genes, including CLDN2 [23], CLDN3 [24], and CLDN14 [25], promote CRC cell proliferation, migration, invasion, and metastasis. However, CLDN6, CLDN7, CLDN18, and CLDN23 have been reported to inhibit CRC progression [26,27,28,29]. Herein, CLDN1 expression was increased in CRC patients, and elevated CLDN1 levels were strongly correlated with reduced overall survival. This finding aligns with prior studies that have established CLDN1 as a promoter of CRC aggressiveness and a prognostic biomarker [30,31]. Mechanistically, CLDN1-driven tumor progression has been associated with increased cell proliferation, invasion, and migration [30]. The terms proliferation, migration, and invasion are fundamental concepts in biology, especially within developmental biology, wound healing, and cancer biology [32]. They are often closely linked and coordinated, especially in pathological contexts such as cancer metastasis [33]. Understanding their intricate crosstalk is essential for elucidating developmental and healing processes and for advancing the development of novel anticancer therapies. Our functional studies revealed that CLDN1 knockdown in CRC significantly attenuated cell viability, migration, and invasion. Moreover, CLDN1 overexpression in xenograft models potentiated tumor growth *in vivo*, as evidenced by decreased tumor volume and weight. This finding aligns with a previous study indicating that targeting CLDN1 with an anti-CLDN1 mAb inhibits tumor growth and metastasis in xenograft models [31].

The CD81 signaling pathway has been well characterized as an oncogenic driver in solid tumors, particularly hepatocellular carcinoma and breast cancer [20,34]. High CD81 expression serves as a prognostic indicator, correlating with an increased risk of adverse survival outcome in cancer patients [35], whereas genetic ablation of CD81 in murine models substantially attenuates tumor growth and metastatic potential [13,36], underscoring its critical role in malignant progression. Our data also revealed that CRC cells with CD81 knockdown exhibited reduced migration and invasion characteristics, whereas CD81 overexpression promoted CRC progression.

The ubiquitin‒proteasome system enables posttranscriptional protein modification and is a major pathway for the degradation of most proteins in eukaryotic cells. Emerging evidence indicates that GRAIL regulates CD81 turnover by inducing K48-linked polyubiquitination [37], while cell surface CD81 is targeted for degradation via K29- and K63-linked polyubiquitin conjugation [38]. In the current study, CLDN1 interacted with CD81 and inhibited CD81 ubiquitination in CRC cells, thereby increasing CD81 protein expression. Our findings reveal a previously unrecognized mechanism whereby CLDN1 acts as a molecular stabilizer of CD81, subverting its posttranslational regulation to fuel CRC progression. While CLDN1 and CD81 were previously identified as co-facilitators of hepatic C virus infection [39], our study provides the first evidence of their functional collaboration in CRC pathogenesis. This CLDN1-CD81 stabilization axis represents a potential therapeutic target of CRC.

Analysis of CRC patient samples revealed that high CLDN1-CD81 coexpression positively correlated with advanced pathological TNM stage and a higher tumor grade. Furthermore, this coexpression signature showed a significant negative correlation with patient survival and was associated with metastatic risk, highlighting its potential as a metastatic and prognostic biomarker. Therapeutic approaches designed to reduce CLDN1 expression and suppress tumor metastasis have been explored in recent years, including gene therapy [40] and anti-CLDN1 mAbs [31]. In the current study, the anti-CD81 mAb 5A6 effectively abrogated the CLDN1-overexpressing CRC cell invasion and migration *in vitro* and inhibited tumor growth in xenograft models. These findings aligned with those of prior studies demonstrating that targeting CD81 with 5A6 suppresses tumor growth and lung metastasis in xenograft models [20,21]. Collectively, our findings suggest that targeting the CLDN1/CD81 axis by inhibiting CLDN1, neutralizing CD81, or using a combination strategy represents a promising therapeutic approach for CRC.

### 4.2 Limitations

Several important questions remain unanswered. First, the precise binding domains mediating the CLDN1-CD81 interaction must be mapped in detail, as this information could facilitate the development of targeted inhibitors. Second, the ubiquitin type (K48/K29/K63) for CD81 remains to be further explored. Third, the specific E3 ubiquitin ligases responsible for CD81 degradation in the absence of CLDN1 remain unidentified. While our findings demonstrate that CLDN1 modulates CD81 protein levels, further studies are required to determine whether CLDN1 stabilizes CD81 directly or via indirect signaling pathways. It remains unclear whether CLDN1 regulates the ubiquitin-mediated degradation of CD81 via the lysosome or proteasome pathway. Although our study demonstrates the involvement of the anti-CD81 mAb 5A6 in modulating CLDN1-driven CRC progression, the potential contributions of other CD81 interaction partners need to be elucidated. The downstream signaling pathways activated by stable CD81 in CRC cells warrant further investigation. Finally, the functional collaboration between CLDN1 and CD81 in the progression of other cancers remains elusive and can be further explored.

### 4.3 Conclusion

In conclusion, our study demonstrates that CLDN1 knockdown impairs the migration and invasion of CRC cells. Furthermore, the anti-CD81 mAb 5A6 effectively counteracted CLDN1-driven cell invasion and migration *in vitro* and significantly suppressed tumor growth in xenograft models. CLDN1 may regulate CRC progression by mediating the ubiquitination-dependent degradation of CD81. These findings not only advance our understanding of CRC pathogenesis but also highlight the CLDN1-CD81 interaction as a potential therapeutic target for this lethal disease.

## Data Availability

The datasets used and/or analyzed during the current study are available from https://www.jianguoyun.com/p/DW3liGgQuaiFChi81oAGIAA.

## Supplementary Materials

The supplementary material is available online at https://www.techscience.com/doi/10.32604/or.2026.075185/s1.

## Abbreviation

CLDN1	claudin-1
CRC	colorectal cancer
mAb	monoclonal antibody
EMT	epithelial‒mesenchymal transition
AMPK	adenosine 5′-monophosphate-activated protein kinase
TGF-β	transforming growth factor-beta
PI3K	phosphoinositide 3-kinase
AKT	protein kinase B
mTOR	mammalian target of rapamycin
STAT1	signal transducer and activator of transcription 1
ULK1	Unc-51-like autophagy activating kinase 1
ERK	extracellular signal-regulated kinase
JNK	c-Jun N-terminal kinase
STRING	search tool for recurring instances of neighboring genes
IHC	immunohistochemistry
Co-IP	coimmunoprecipitation
TCGA	The Cancer Genome Atlas
COAD	colon adenocarcinoma
FBS	fetal bovine serum
shRNA	short hairpin RNA
CCK-8	Cell Counting Kit-8
qRT‒PCR	quantitative real-time polymerase chain reaction
GAPDH	glyceraldehyde-3-phosphate dehydrogenase
SDS‒PAGE	sodium dodecyl sulfate‒polyacrylamide gel electrophoresis
