# Supplementary material for: Claudin-1 Interacts with CD81 and Promotes the Progression of Colorectal Cancer
Source: Oncol Res. 2026 May 21;34(6):30. doi: 10.32604/or.2026.075185 (PMC13223189; doi:10.32604/or.2026.075185)
Supplement: Supplementary file 1 [file OncolRes-34-75185-s001.zip › TSP_OR_75185-s001.docx]

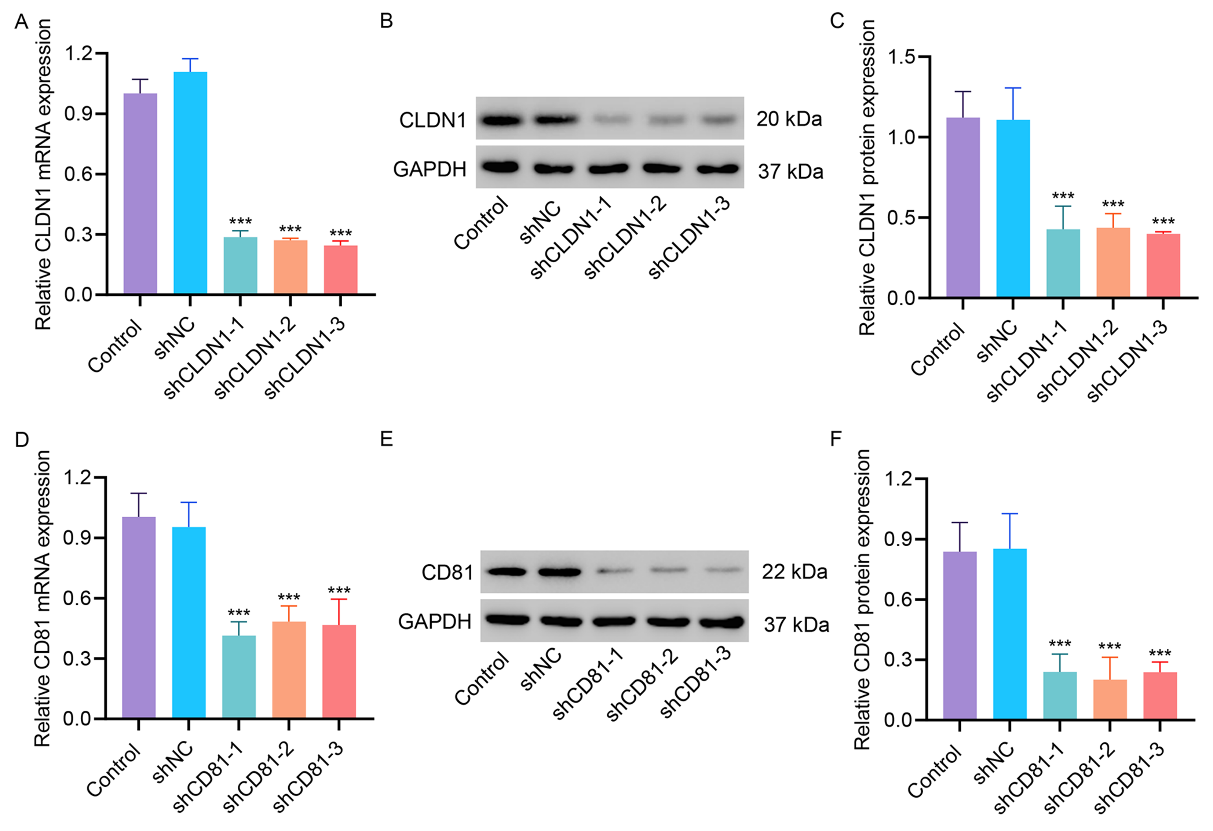


**Figure S1: Claudin-1 (CLDN1) and CD81 silencing in HT29 cells.** Expression levels of (A–C) CLDN1 and (D–F) CD81 in HT29 cells transduced with CLDN1 shRNA or the shNC lentiviral vector. ****P* < 0.001 vs. shNC.
